# Impact of bacteroides uniformis on fatty liver hemorrhagic syndrome in dawu golden phoenix laying hens: modulation of gut microbiota and arachidonic acid metabolism

**DOI:** 10.3389/fmicb.2025.1560887

**Published:** 2025-04-28

**Authors:** Yu Zhang, Rongfei Ma, Xicui Du, Xin He, Yan Zhang, Ning Ma, Hailong Liu, Xinghua Zhao

**Affiliations:** ^1^College of Veterinary Medicine, Hebei Agricultural University, Baoding, China; ^2^Hebei Jinkun Animal Pharmaceutical Co. Ltd., Xinji, China; ^3^Institute of Animal Science and Veterinary Medicine, Hainan Academy of Agricultural Sciences, Haikou, China

**Keywords:** fatty liver hemorrhagic syndrome, laying hens, *Bacteroides uniformis*, gut microbiota, arachidonic acid

## Abstract

This study explored the impact of *Bacteroides uniformis* (*B. uniformis*) on fatty liver hemorrhagic syndrome (FLHS) induced by a high-energy and low-protein (HELP) diet in laying hens, mainly focusing on hepatic lipid metabolism, gut microbiota, and arachidonic acid (AA) metabolism. A total of 120 Dawu Golden Phoenix laying hens (210-day-old) were randomly divided into four groups. The control group (CON) was fed a standard diet and received a daily gavage of PBS, while the other groups were fed with a HELP diet to induce FLHS and received a daily gavage of PBS (MOD), 1 × 10^9^ CFU/ml *B. uniformis* (BUL), and 1 × 10^11^ CFU/ml *B. uniformis* (BUH) for 70 days. All hens were administered 1 ml daily by gavage. Each group had 6 replications with 5 hens per replication. The results showed that *B. uniformis* increased the egg production rate and feed conversion ratio and decreased body weight, liver index, and abdominal fat rate (*p* < 0.05). *B. uniformis* treatment reduced liver lipid accumulation by reducing the levels of Triglyceride (TG), Total cholesterol (TC), low-density lipoprotein cholesterol (LDL-C), alanine transaminases (ALT), and aspartate transaminases (AST) in serum and significantly elevated high-density lipoprotein cholesterol (HDL-C) (*p* < 0.05). The results indicated that *B. uniformis* altered the gut microbiota. Specifically, the abundance of *Bacteroides* was higher, and the relative abundances of *Treponema, Helicobacter,* and Spirochaetota were lower than those of the MOD group (*p* < 0.05). Moreover, targeted metabolomic analysis showed that supplementation of *B. uniformis* significantly elevated 6-keto-PGF_1α_ and AA levels, along with significantly reduced levels of thromboxane B_2_ (TXB_2_), leukotriene D_4_ (LTD_4_), 8-isoprostaglandin F_2α_ (8-iso-PGF_2α_), 12S-hydroxyeicosatetraenoic acid (12S-HETE), 15S-hydroxyeicosatetraenoic acid (15S-HETE), 9-S-hydroxy-octadecadienoic acid (9S-HODE), and 13-S-hydroxy-octadecadienoic acid (13S-HODE) (*p* < 0.05). In conclusion, the oral intake of *B. uniformis* can improve liver function, gut microbiota, and AA metabolism, thereby helping to ameliorate FLHS in Dawu Golden Phoenix laying hens.

## 1 Introduction

The widespread use of cage rearing in the laying hen industry, coupled with restricted movement, increased production pressures, and fluctuating temperatures, adversely affects hepatic lipid metabolism, making laying hens more prone to fatty liver hemorrhagic syndrome (FLHS) (Yadav et al., 2024). In a survey, FLHS accounted for 74% of the total mortality rates in caged laying hens in Queensland (Shini et al., 2018; Anene et al., 2023). The main pathological characteristics of FLHS are hepatic steatosis and, in severe cases, vascular rupture that can lead to sudden death from hemorrhage (Anene et al., 2023). Studies have shown that high-energy and low-protein (HELP) diets are the fundamental pathological contributors to FLHS (Yaqoob et al., 2024). The occurrence of FLHS is first caused by the excessive accumulation of triglycerides (TG) in the liver, inhibition of fatty acid oxidation, and disorders in liver lipid metabolism. Subsequently, insulin resistance, liver oxidative stress, and liver inflammation can further aggravate FLHS (Cheng et al., 2024). Laying hens affected by FLHS closely mimic the conditions seen in humans with non-alcoholic fatty liver disease (NAFLD), given that their livers are a major site for *de novo* lipogenesis (Gao et al., 2021). These findings render them a suitable model for investigating human lipid metabolism disorders. Consequently, using laying hens in research provides a novel and promising avenue for deepening our understanding of NAFLD pathophysiology and exploring potential therapeutic strategies.

Changes to the intestinal microbiota’s composition and functionality can significantly influence the development and advancement of FLHS (Li et al., 2020). Due to their low cost and high safety profile, probiotics have emerged as an ideal option for attenuating liver lipid metabolism disorders caused by high-energy diets (Wang et al., 2022). In particular, *B. uniformis* has been extensively used in clinical settings due to its safety and excellent efficacy. It has been reported that *B. uniformis* is inversely associated with serum LDL-cholesterol levels (Yan et al., 2022). Furthermore, *B. uniformis* has been documented to prevent obesity and metabolic disorders (Gómez del Pulgar et al., 2020; Fabersani et al., 2021; Lee et al., 2021; López-Almela et al., 2021). Currently, there is a lack of comprehensive studies on the effects of *B. uniformis* on lipid metabolism in laying hens, particularly regarding the treatment of FLHS induced by high-energy diets. Arachidonic acid (AA), a polyunsaturated fatty acid, is ubiquitous in living organisms (Pereira et al., 2024). It has been confirmed that a disorder in AA metabolism, characterized by reduced levels of AA, is associated with FLHS (Meng et al., 2021). Previous studies have shown that *Bacteroides fragilis* exerts an anti-FLHS effect by modulating oxylipin metabolism and gut microbiota stability, with AA playing a pivotal role (Zhang et al., 2024a, 2024b). Therefore, we hypothesized that *B. uniformis* could effectively relieve FLHS in laying hens by regulating AA metabolism and gut microbiota.

This study aims to elucidate the improvement effect of *B. uniformis* on FLHS by examining liver lipid metabolism, gut microbiota, and AA metabolism and provide a basis for its application in laying hens.

## 2 Materials and methods

### 2.1 Materials

The *Bacteroides uniformis* strain was generously provided by Dr. Ma Ning from Hebei Agricultural University (Hebei, China). This bacterial strain was extracted from the cecal contents of healthy laying hens. By aligning its 16S rRNA gene sequence with the NCBI reference database,^1^ the strain was confirmed to be *Bacteroides uniformis* strain 19006C (Supplementary Figure S1). All chemicals and solvents were obtained from commercial suppliers and were used as received.

### 2.2 Bacterial strains and culture conditions

The details of bacterial strains and culture conditions are provided in the Supplementary material.

### 2.3 Bile acid and simulated gastrointestinal fluid tolerance

The stress resistance of *B. uniformis* is detailed in the Supplementary material.

### 2.4 Animals and treatment

We acquired 210-day-old 120 Dawu Golden Phoenix laying hens from Hebei Huafeng Farm (Baoding, Hebei, China). The laying hens were housed in wire cages (measuring 100 cm × 70 cm × 60 cm) with five hens per cage. They had free access to clean water and were fed twice daily (7:00 a.m. and 5:00 p.m.). The room temperature was maintained at 25 ± 1°C, with the humidity level of 50 ± 5%. The formulation of the standard diet adhered to the guidelines of the Chinese Feeding Standard of Chickens NPC (1994). The detailed composition and nutritional levels of the standard and HELP diets are presented in Supplementary Table S1. The animal experiment protocol (No. 2022161) was reviewed and approved by the Animal Protection and Ethics Committee of Hebei Agricultural University.

All laying hens were allowed to adapt for 1 week before the experiment and were randomly allocated into four treatment groups, with each group consisting of six replications (five hens per replication). The experimental design was as follows: The control group (CON) was fed a standard diet and given a daily gavage of phosphate-buffered saline (PBS), whereas the other three groups were all fed with a HELP diet to induce FLHS (Zhang et al., 2024a, 2024b). Among the three FLHS groups, one was given a daily gavage of PBS (MOD), and the other groups were treated with 1 × 10^9^ CFU/ml *B. uniformis* (BUL) and 1 × 10^11^ CFU/ml *B. uniformis* (BUH). All hens were given 1 ml daily by gavage. The experiment lasted for 70 days (Table 1).

**Table 1:** Experimental treatment.

Groups	Diet	Treament (once daily gavage, 70 days)
CON	Standard diet	1 ml PBS/hen
MOD	HELP diet	1 ml PBS/hen
BUL	HELP diet	1 mL 1 × 10^9^ CFU/ml *B. uniformis*/hen
BUH	HELP diet	1 mL 1 × 10^11^ CFU/ml *B. uniformis*/hen

### 2.5 Measurements and sample collection

The body weights of the laying hens were measured every 2 weeks. Daily feed intake and residual feed were recorded to calculate the average daily feed intake (ADFI). Eggs were collected and weighed daily. The egg-laying rate was calculated based on egg production data on a hen-day basis. The feed conversion ratio (FCR) was determined as grams of feed per gram of egg produced. At the end of the animal experiment, the laying hens underwent a 12-h fasting. Blood samples were collected from the brachial vein and centrifuged (3,500 r/min, 15 min) to obtain serum at 4°C. The serum was then stored at −20°C for further analysis. Subsequently, the laying hens were sacrificed by cervical dislocation and exsanguinated, and the livers and abdominal fat were carefully removed and weighed. A piece of liver tissue was immersed in a 10% formalin solution. The remaining liver tissue and cecal contents were immediately frozen in liquid nitrogen and stored at −80°C until further targeted metabolomic analysis and bacterial DNA extraction.

### 2.6 Production performance and liver index

At the end of the animal experiment (the 70^th^ day), the egg production rate was calculated according to Equation 1 (Xiong et al., 2024). The ADFI was calculated according to Equation 2 (Taofeek et al., 2022). The FCR was calculated according to Equation 3 (Al-Khalaifah et al., 2024). The liver index was calculated according to Equation 4 (Zhang et al., 2024a, 2024b). The abdominal fat rate was calculated according to Equation 5 (Zhao et al., 2020).


(1) $$\mathrm{Egg}\;\mathrm{production rate}\;\left(\%\right)=\frac{\mathrm{number of eggs laid}}{\mathrm{number of laying hens}}\times 100\%$$

(2) $$\mathrm{ADFI}\;\left(g/d\right)=\frac{\mathrm{overall feed intake}-\mathrm{overall residual feed}}{\mathrm{days}\times \mathrm{number of laying hens}}\times 100\%$$

(3) $$FCR=\frac{\mathrm{overall feed intake}-\mathrm{overall residual feed}}{\mathrm{overall weight of eggs}}\times 100\%$$

(4) $$\mathrm{Liver index}\;\left(\%\right)=\frac{\mathrm{weight of the liver}}{\mathrm{body weight}}\times 100\%$$

(5) $$\mathrm{Abdominal}\;\mathrm{fat}\;\mathrm{rate}\;\left(\%\right)=\frac{\mathrm{weight of abdominal}\;\mathrm{fat}}{\mathrm{body weight}}\times 100\%$$

### 2.7 Biochemistry analysis and liver morphology

Total cholesterol (TC), TG, low-density lipoprotein cholesterol (LDL-C), high-density lipoprotein cholesterol (HDL-C), aspartate transaminases (AST), and alanine transaminases (ALT) were measured in serum following the instructions provided in the kit (Nanjing Jiancheng Institute of Bioengineering, Jiangsu, China). After formalin fixation, the liver tissues were embedded in paraffin and sectioned into 5-μM slices. These sections were subsequently stained with standard hematoxylin and eosin (H & E) for histological examination. Additionally, frozen liver sections were stained with Oil Red O to assess lipid droplet accumulation.

### 2.8 16S rRNA gene sequencing analysis

The bacterial DNA of the cecal samples was extracted using the Cetyltrimethylammonium Bromide method, following the barcode sequence. The Polymerase Chain Reaction (PCR) amplification primer sequence split the sample data from the offline data, and the forward primer was 515F (5′-CCTAYGGGRBGCASCAG-3′) with reverse primer 806R (5′-GGACTACNNG GGTATCTAAT3′), which amplified the variable region 4 of the microbial 16S rDNA gene. The NEBNext® Ultra™ II FS DNA PCR-free Library Prep Kit (New England Biolabs (Beijing) LTD, Beijing, China) was used to construct the library. The constructed library was quantified using Qubit and qPCR. After the library was qualified, PE 250 on-machine sequencing was performed using a NovaSeq 6000 platform (Illumina Corporation, CA, USA).

Fast Length Adjustment of SHort reads (FLASH Version 1.2.11) was used to assemble the reads of the sample to obtain raw tags, and fastp software (Version 0.23.1) was used for quality control to obtain clean tags. The clean tags remove chimeric sequences by comparing them to the Silva database. Taxonomic annotation and analysis were conducted at both the phylum and genus levels, using the Silva 138.1 database. To assess microbial community diversity, alpha diversity analysis was conducted using Quantitative Insights Into Microbial Ecology 2. Beta diversity analysis utilized the weighted UniFrac distance algorithm and was visualized through principal coordinate analysis (PCoA) plots. The independent T-test was used to analyze inter-group differences in species abundance at various taxonomic levels, focusing on those with statistically significant variations. Linear discriminant analysis effect size (LEfSe) analysis was performed using the microeco package (Linear discriminant analysis (LDA) score > 2). Spearman’s correlation analysis was performed to explore the relationships between differential bacteria, serum lipids, and AA metabolites.

### 2.9 Targeted metabolomics analysis

To determine the changes in AA metabolites in the CON, MOD, and BUL layers, quantitative analysis of AA metabolites in the liver was performed using targeted metabolomics.

The reagents, including methanol, acetonitrile, acetic acid, and isopropanol, were sourced from Merck (NJ, USA). Standard substances and internal standards were procured from Cayman (MI, USA). Liver tissue samples were ground in liquid nitrogen. A total of 20 mg of liver tissue powder was extracted using 200 μl of methanol. The samples were vortexed, and the protein precipitate was allowed to settle at 4°C. Furthermore, 20 μl of an internal standard mixture (1 μM) was added to each sample and vortexed thoroughly. The samples were then centrifuged at 5,000 × *g* for 10 min at 4°C. The extraction process was repeated, and the supernatants from both extractions were combined. Oxylipins were extracted using solid-phase extraction columns. After the concentration and drying of the elution solution, reconstitution was performed using 100 μl of methanol/water (1:1, *v*/*v*) for LC–MS/MS analysis.

The chromatographic conditions were as follows: the samples were separated using an Agilent 1,290 Infinity LC ultra-high-performance liquid chromatography system (Agilent Technologies Inc., CA, USA). The separation of samples was performed on ACQUITY UPLC BEH C18 (1.7 μm, 2.1 mm × 50 mm, Waters, Ireland) at a flow rate of 400 μl/min. Mobile phase A consisted of a 0.1% formic acid aqueous solution, and mobile phase B consisted of a 0.1% formic acid acetonitrile solution. The mobile phase gradient was as follows: 0 to 1 min, 30% B; 1 to 9 min, 30 to 90% B; 9 to 11 min, 90% B; 11 to 11.1 min, 90 to 20%; and 11.1 to 14 min, 20% for equilibration. The injection volume was 20 μl, the column temperature was 35°C, and the autosampler temperature was 4°C.

The mass spectra data were acquired by using a 5,500 QTRAP mass spectrometer (SCIEX, MA, USA) with an ESI source operated in negative mode. The electrospray Ionization parameters were optimized as follows: source temperature, 500°C; Ion Source Gas1, 50; Ion Source Gas2, 50; Curtain gas, 30; IonSapary Voltage Floating, and −4,500 V. The ion pairs to be tested were detected using the multiple reaction monitoring mode.

Multiquant 3.0.2 software (SCIEX, MA, USA) was used to extract chromatographic peak areas and retention times. The standard of the target substance was used to correct the retention time for metabolite identification.

### 2.10 Statistical analysis

All data were analyzed using SPSS 27.0 software (IBM Inc., NY, USA). Shapiro–Wilk and Levene’s tests were used to assess the normal distribution of the data and the homogeneity of variance. One-way analysis of variance (ANOVA) and Tukey’s *post hoc* test were employed to determine the significance of mean differences. The results are expressed as mean ± SEM, and a significant difference was set as *p* < 0.05. The data were plotted through Origin software (OriginPro 2021, OriginLab Corporation, USA).

## 3 Results

### 3.1 *Bacteroides uniformis* stress resistance

The survival rate of *B. uniformis* in BHI broth at pH 3 was 32.10%. Furthermore, the survival rates of *B. uniformis* in 0.1, 0.3, and 0.5% bile salts were 110.33, 63.04, and 51.16%, respectively. Additionally, the survival rates of *B. uniformis* in simulated gastric juice and intestinal juice were 14.61 and 8.16%, respectively (Table 2).

**Table 2:** *B. uniformis* stress resistance.

Treatment		The survival rate (%)
Hydrochloric acid (pH = 3, 3 h)		32.10 ± 1.74
Sodium Taurocholate (12 h)	0.1%	110.33 ± 5.75
	0.3%	63.04 ± 2.37
	0.5%	51.16 ± 4.60
Gasttic juice (4 h)		14.61 ± 0.95
Intestinal juice (4 h)		8.16 ± 1.58

The data was expressed as mean ± SD.

### 3.2 Effects of *B. uniformis* on the production performance of FLHS-laying hens

**Figure 1:**
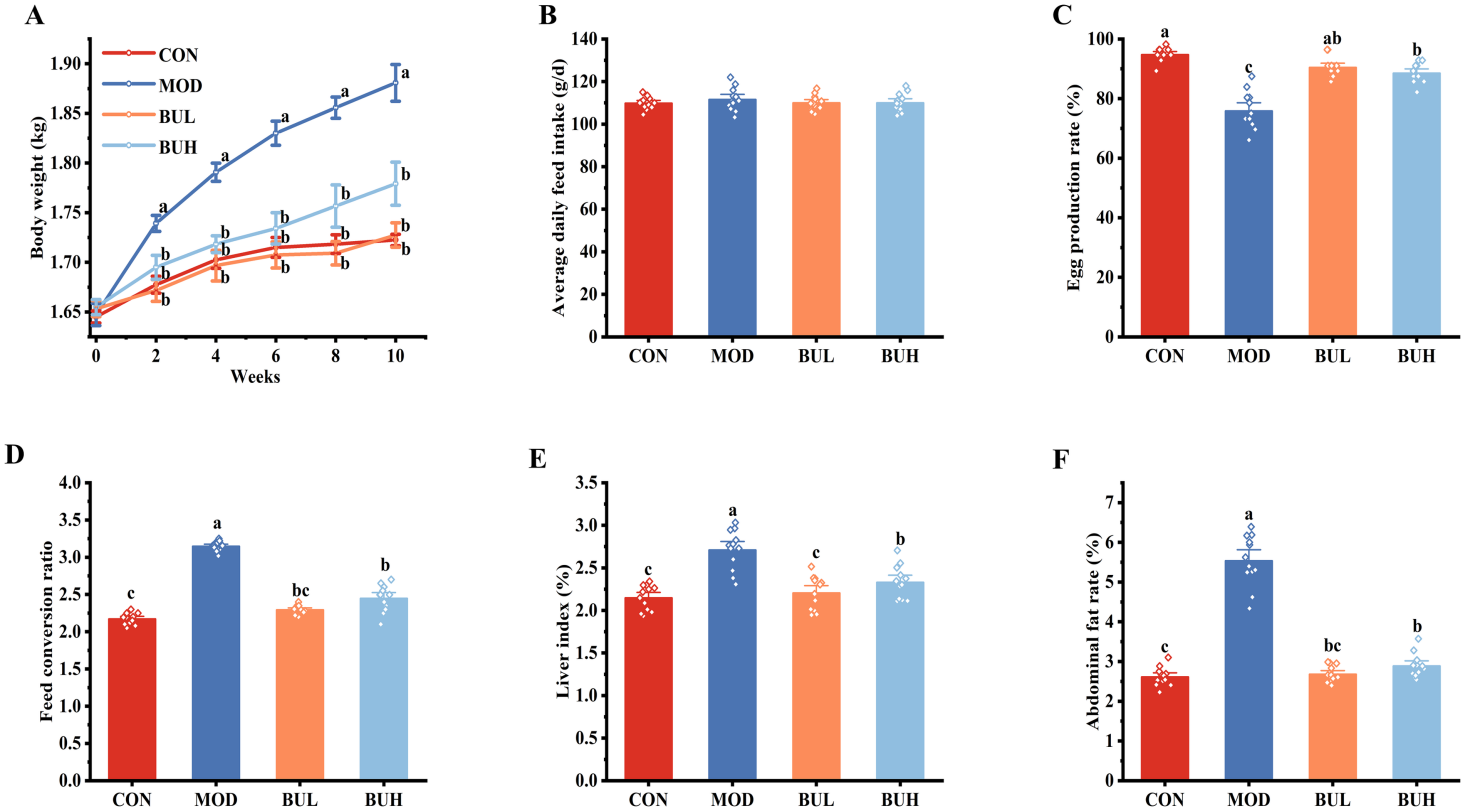
Effect of *B. uniformis* on production performance and liver index. **(A)** Body weight. **(B)** Average daily feed intake. **(C)** Egg production rate. **(D)** Feed conversion ratio. **(E)** Liver index. **(F)** Abdominal fat rate. Means with different superscripts differ significantly at a significance level of *p* < 0.05, *n* = 12.

As can be seen from Figure 1A, the body weight of the MOD group was significantly higher than that of other groups from week 2 (*p* < 0.05). A slight improvement in body weight was observed in laying hens of the BUH group, but no statistically significant differences were observed between the CON, BUL, and BUH groups (*p* > 0.05). In terms of average daily feed intake, there was no significant difference between all laying hens in the four groups (*p* > 0.05) (Figure 1B). The egg production rate of the MOD group was significantly lower than that of the other three groups (*p* < 0.05) (Figure 1C). In addition, the egg production rates of the MOD group and BUH group were significantly lower than those of the CON group (*p* < 0.05). Interestingly, the egg production rates of the BUH and BUL groups were significantly higher than those of the MOD groups during the experiment (*p* < 0.05). The egg production rates of the CON, BUL, BUH, and MOD groups were 94.79, 90.48, 88.54, and 75.89%, respectively. The FCR of the BUH and BUL groups was significantly lower than that of the MOD group (*p* < 0.05) (Figure 1D). Upon closer inspection, we also discovered that the FCR of the BUL group was statistically similar to that of the CON group. Details of egg production, egg weight, liver weight, and abdominal fat weight are shown in Supplementary Table S2.

### 3.3 Effects of *B. uniformis* on liver index and liver morphology in FLHS-laying hens

The liver index and abdominal fat rate of the MOD group were significantly higher than the CON group (*p* < 0.05) (Figures 1E,F). In addition, the liver index and abdominal fat rate were significantly reversed following the administration of *B. uniformis* (*p* < 0.05) (Figures 1E,F). Notably, the reversal effect on the liver index was significantly better in the BUL group than in the BUH group (*p* < 0.05).

**Figure 2:**
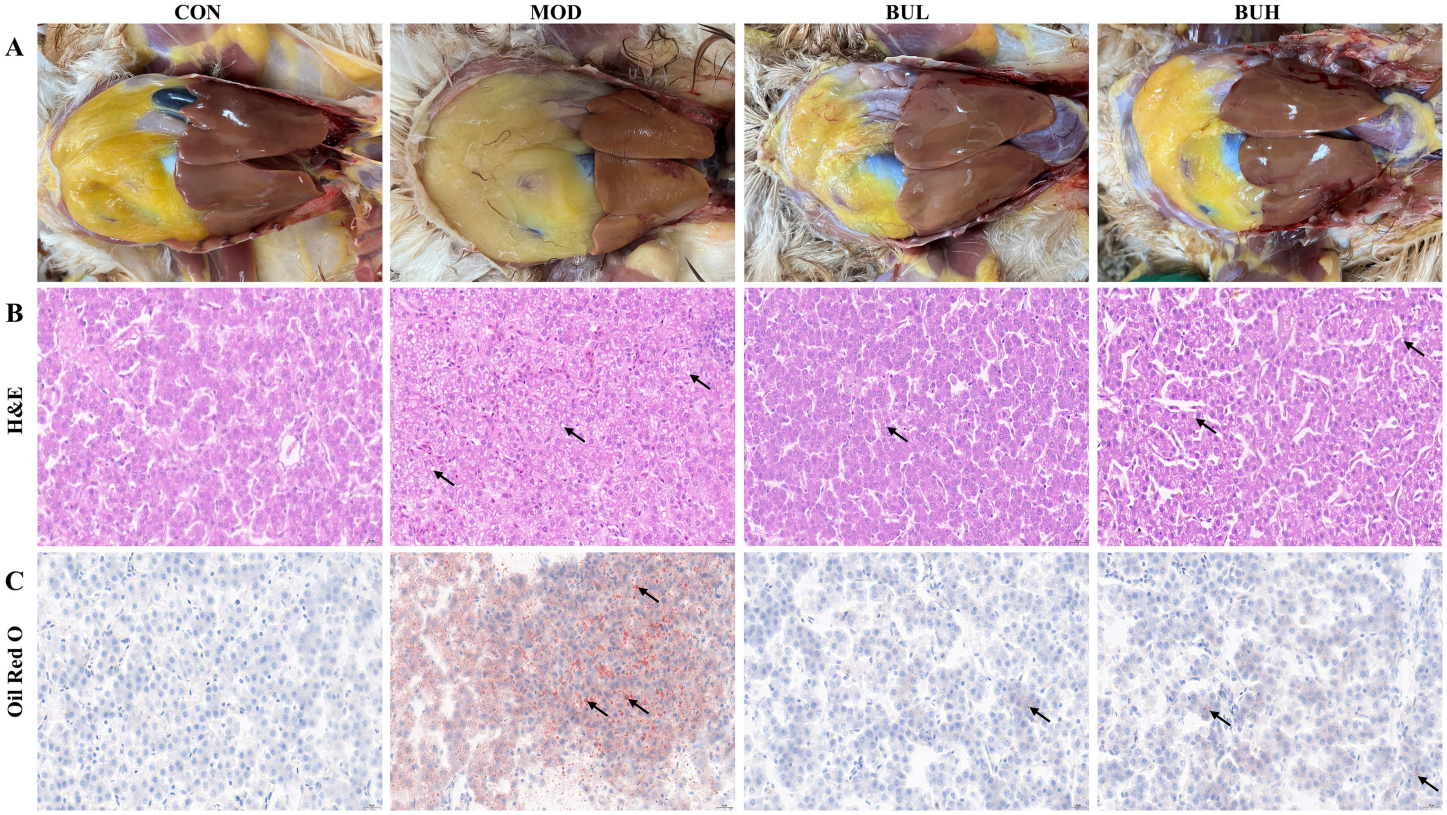
Effect of *B. uniformis* on liver morphology of FLHS-laying hens. **(A)** Anatomical map of the liver. **(B)** Liver tissues from the CON, MOD, BUL, and BUH groups were subjected to H&E staining (scale bar: 20 μm). **(C)** Liver tissues from the CON, MOD, BUL, and BUH groups were subjected to Oil Red O staining (scale bar: 20 μm).

The results of pathological examination of the liver staining with H & E and Oil Red O demonstrated a clear and normal liver cell architecture in the CON group. Serious fatty degeneration with lipid droplet accumulation and necrosis was found in laying hens with the HELP diet in the MOD group. In comparison with the MOD group, HELP diet-induced lipid accumulation in the liver of laying hens with *B. uniformis* treatment was decreased at different degrees, indicating that the fatty degeneration was ameliorated by *B. uniformis* treatment (Figure 2).

### 3.4 Effects of *B. uniformis* on serum lipid levels in FLHS-laying hens

**Figure 3:**
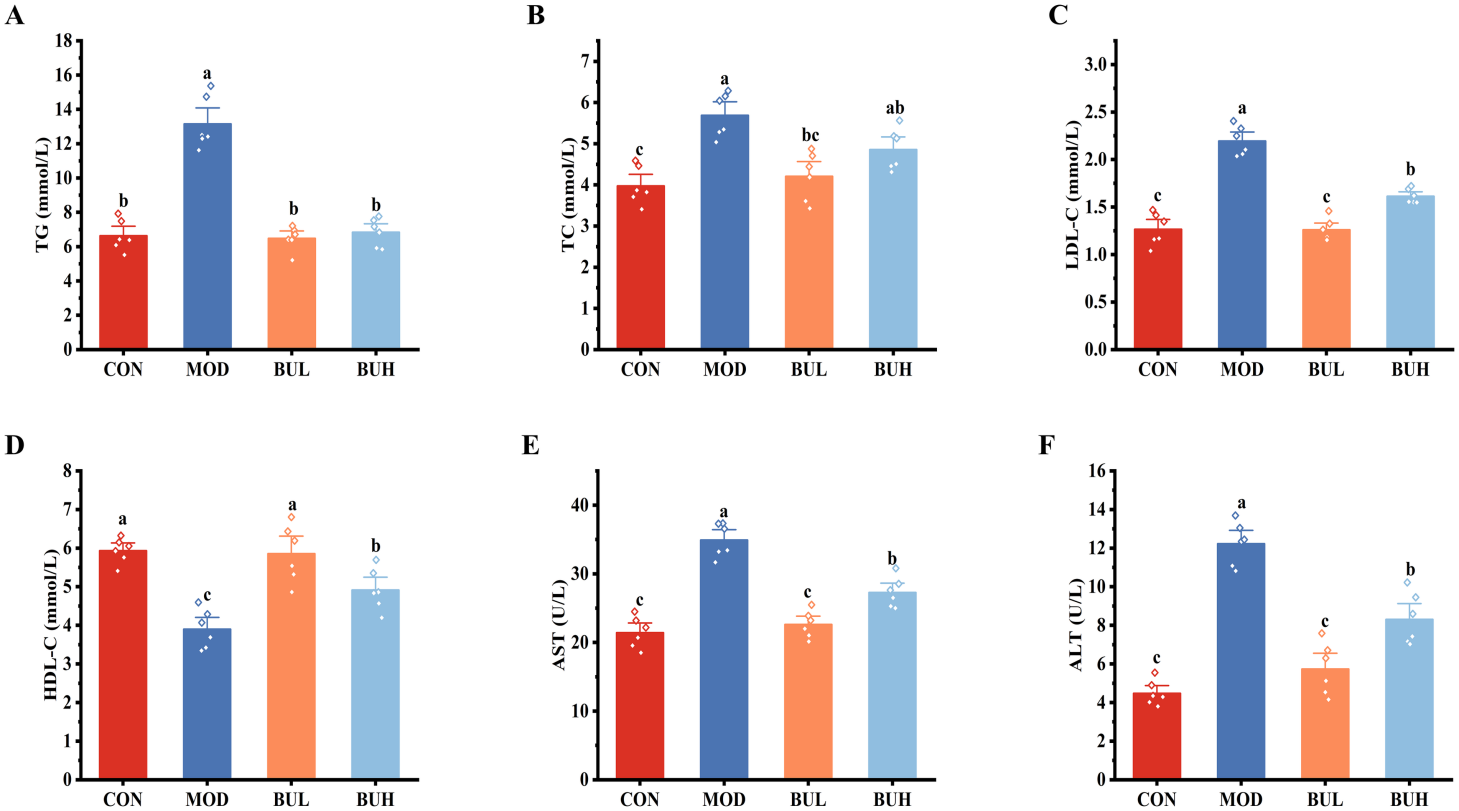
Effect of *B. uniformis* on blood lipids and liver function of FLHS-laying hens. **(A–F)** TG, TC, LDL-C, HDL-C, AST, and ALT, respectively. Means with different superscripts differ significantly at a significance level of *p* < 0.05, *n* = 6.

As shown in Figure 3, the levels of TG, TC, LDL-C, AST, and ALT in the MOD group were significantly increased (*p* < 0.05), whereas HDL-C levels were significantly decreased compared to the CON group (*p* < 0.05). All these indicators were significantly reversed by *B. uniformis* treatment. Notably, the LDL-C, HDL-C, AST, and ALT levels in the BUL group were reversed to a significantly greater extent than those in the BUH group (*p* < 0.05). These results indicated that the reversal effect of BUL was better than that of BUH. Consequently, the BUL group was selected for further experimentation.

### 3.5 Effects of *B. uniformis* on the gut microbiota of FLHS-laying hens

**Figure 4:**
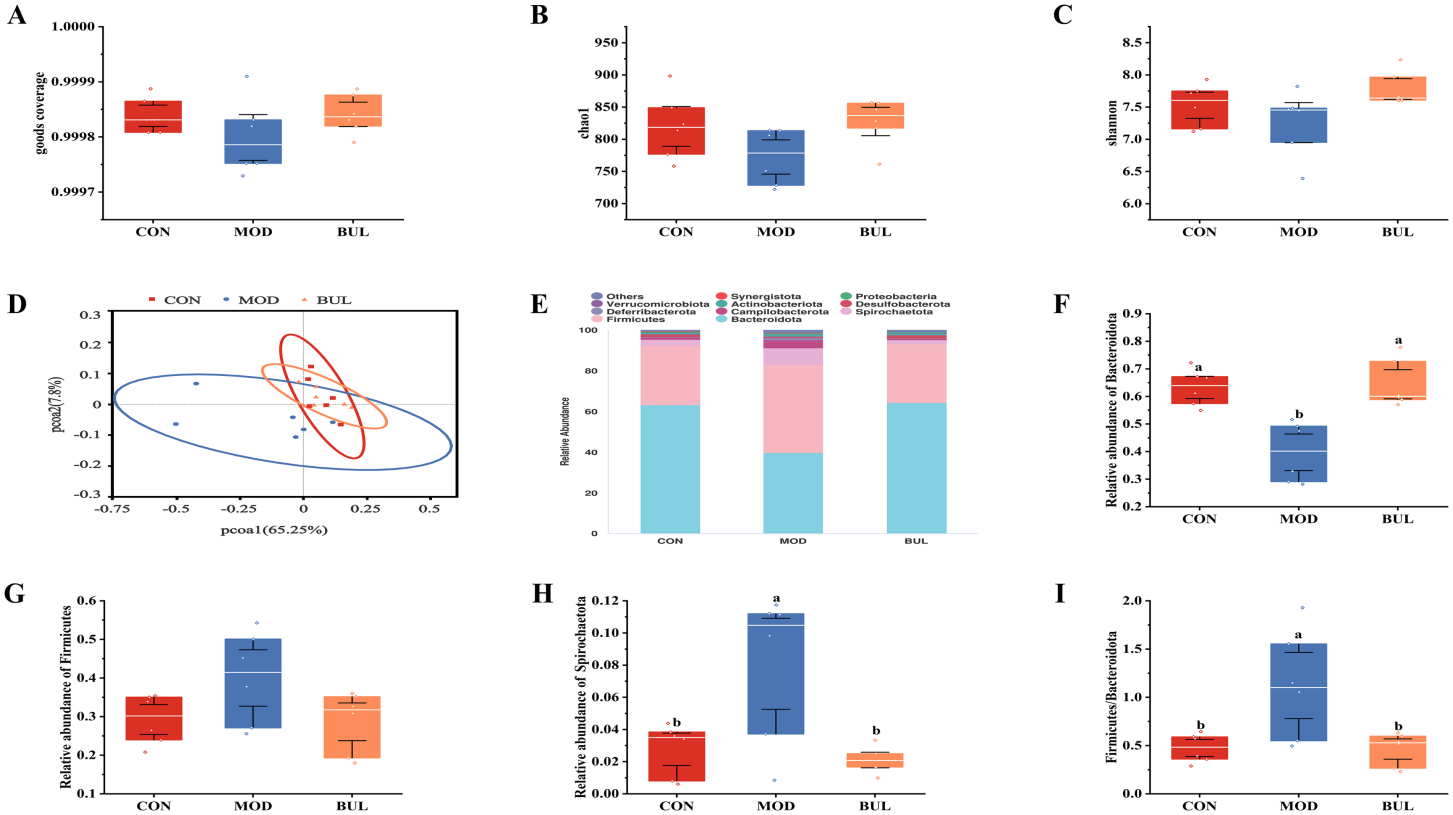
Effect of *B. uniformis* on the phylum-level abundance of gut microbiota in FLHS-laying hens. **(A)** Goods coverage index. **(B)** Chao1 index. **(C)** Shannon index. **(D)** PCoA plot based on weighted_unifrac. **(E)** Relative abundance of gut microbiota at the phylum level. **(F)** Relative abundance of Bacteroidota. **(G)** Relative abundance of Firmicutes. **(H)** Relative abundance of Spirochaetota. **(I)** Firmicutes/Bacteroidetes. Means with different superscripts differ significantly at a significance level of *p* < 0.05, *n* = 6.

**Figure 5:**
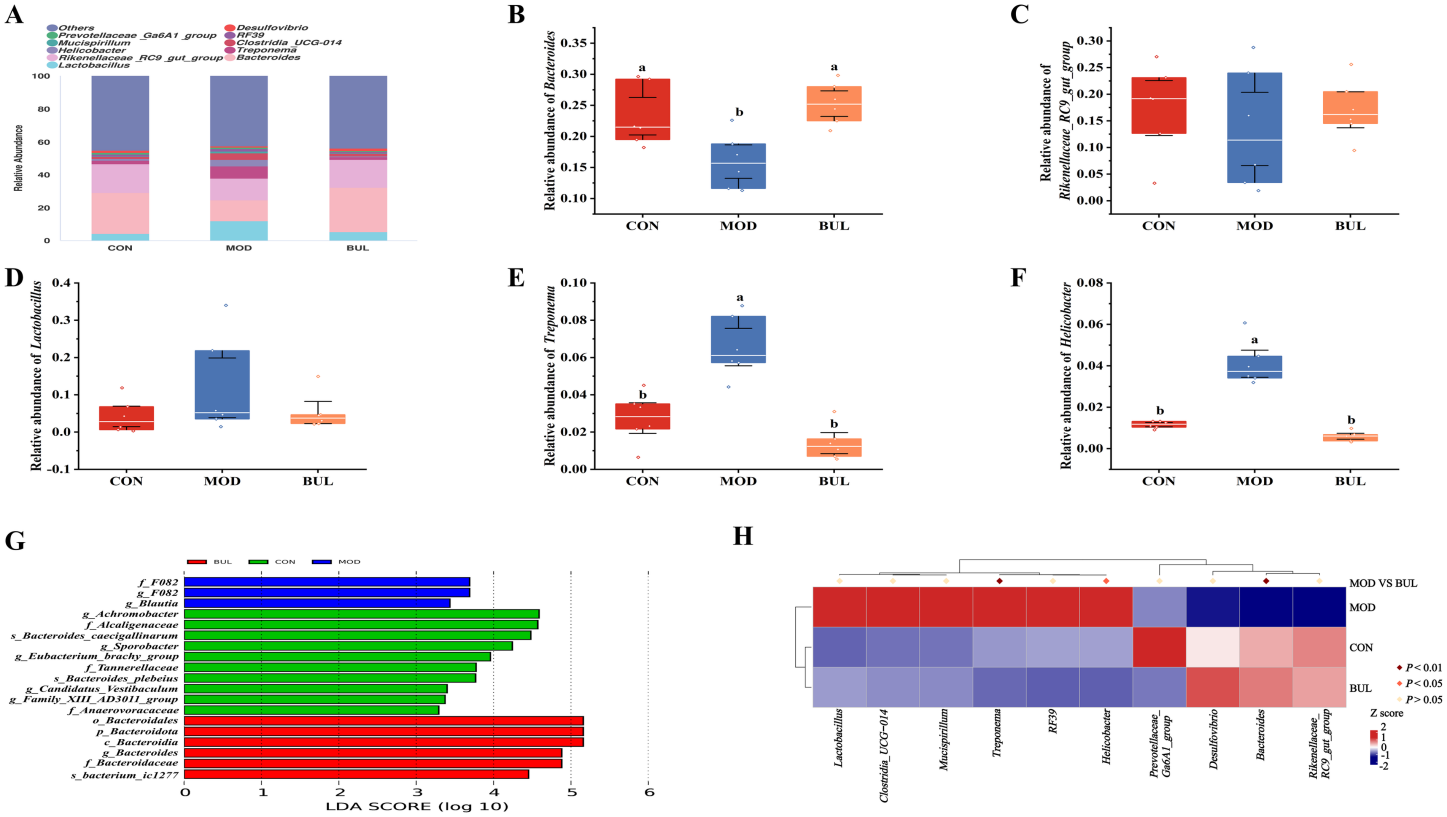
Effects of *B. uniformis* on relative abundance of gut microbiota at the genus level in FLHS-laying hens. **(A)** Relative abundance of gut microbiota at genus level. **(B)** Relative abundance of *Bacteroides*. **(C)** Relative abundance of *Rikenellaceae_RC9_gut_group*. **(D)** Relative abundance of *Lactobacillus*. **(E)** Relative abundance of *Treponema*. **(F)** Relative abundance of *Helicobacter*. **(G)** LEfSe analysis. **(H)** Metastat analysis to identify the contribution of differential genus bacteria. Means with different superscripts differ significantly at a significance level of *p* < 0.05, *n* = 6.

The goods coverage value was close to 1, indicating that the sequencing depth was likely comprehensive enough to encompass nearly all species within the sample (Figure 4A). The BUL group had the highest observed levels of the Chao1 index and Shannon index, but there was no significant difference (*p* > 0.05) (Figures 4B,C). As shown in Figure 4D, the microbial community structure that could reveal distinct clustering of gut content samples in the MOD group was separated from the CON group, whereas the microbial community structure in the BUL group was obviously altered after *B. uniformis* administration, indicating that *B. uniformis* resisted the dysbiosis of the microbiota community induced by HELP in Dawu Golden Phoenix laying hens. At the phylum level, Bacteroidota, Firmicutes, and Spirochaetota were the dominant phyla, with Bacteroidota being the most abundant (Figure 4E). Supplementation with *B. uniformis* significantly decreased relative abundance of Spirochaetota while significantly increasing relative abundance of Bacteroidota (*p* < 0.05). However, there was no significant difference in the relative abundance of Firmicutes among the groups (*p* > 0.05) (Figures 4F–I). At the genus level, *Bacteroides*, *Rikenellaceae_RC9_gut_group*, *Lactobacillus*, *Treponema*, and *Helicobacter* were the dominant genera (Figure 5A). Supplementation with *B. uniformis* significantly decreased the relative abundances of *Treponema* and *Helicobacter* and significantly increased the relative abundance of *Bacteroides* (*p* < 0.05). However, there was no significant difference in the relative abundance of the *Rikenellaceae_RC9_gut_group* and *Lactobacillus* among the groups (*p* > 0.05) (Figures 5B–F). LEfSe analysis confirmed significant enrichment of Bacteroidales, Bacteroidota, Bacteroidia, *Bacteroides*, *and* Bacteroidaceae in the BUL group, identifying them as signature differential bacteria (Figure 5G). The comparative metastat analysis revealed significant differences in the microbial composition between the MOD and BUL groups. Notably, *Treponema* and *Helicobacter* exhibited a marked decrease in the BUL group compared to the MOD group (*p* < 0.05 or *p* < 0.01). In contrast, *Bacteroides* was more abundant in the BUL group (*p* < 0.01), suggesting a potential increase. These findings underscore the distinct microbial signatures associated with the MOD and BUL groups, highlighting the importance of *Bacteroides* in the BUL group’s microbiota profile (Figure 5I).

### 3.6 Effect of *B. uniformis* on AA metabolism in FLHS-laying hens

**Figure 6:**
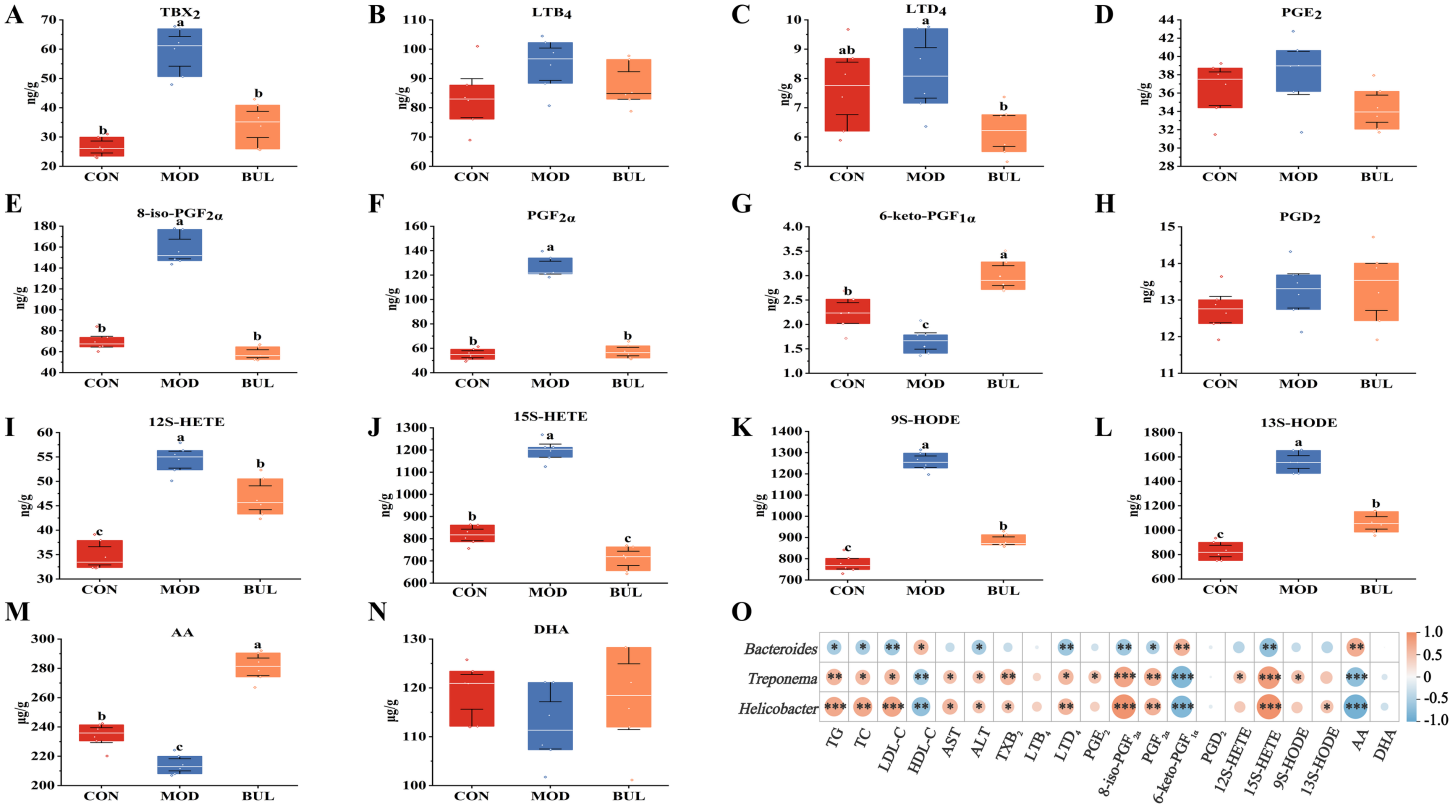
Targeted metabolomics analysis and correlation analysis. **(A)** TXB_2_. **(B)** LTB_4_. **(C)** LTD_4_. **(D)** PGE_2_. **(E)** 8-iso-PGF_2α_. **(F)** PGF_2α_. **(G)** 6-keto-PGF_1α_. **(H)** PGD_2_. **(I)** 12S-HETE. **(J)** 15S-HETE. **(K)** 9S-HODE. **(L)** 13S-HODE. **(M)** AA. **(N)** DHA. **(O)** Correlation analysis. The metabolite units are expressed as ng/g and μg/g liver tissue. Means with different superscripts differ significantly at a significance level of *p* < 0.05, *n* = 6. * indicates *p* < 0.05, ** indicates *p* < 0.01, and *** indicates *p* < 0.001.

To confirm the relationship between AA metabolism and FLHS and the effect of *B. uniformis* on AA metabolism in Dawu Golden Phoenix laying hens, the composition and content of AA metabolites among the three groups were analyzed using targeted metabolomics. In targeted metabolomics analysis, 14 distinct oxidized lipid metabolites were identified. These included thromboxane B_2_ (TXB_2_), leukotriene B_4_ (LTB_4_), leukotriene D_4_ (LTD_4_), prostaglandin E_2_ (PGE_2_), 8-isoprostaglandin F_2α_ (8-iso-PGF_2α_), prostaglandin F_2α_ (PGF_2α_), 6-keto-prostaglandin F_1α_ (6-keto-PGF_1α_), prostaglandin D_2_ (PGD_2_), 12S-hydroxyeicosatetraenoic acid (12S-HETE), 15S-hydroxyeicosatetraenoic acid (15S-HETE), 9-S-hydroxy-octadecadienoic acid (9S-HODE), 13-S-hydroxy-octadecadienoic acid (13S-HODE), AA, and docosahexaenoic acid (DHA). The targeted metabolomics analysis results showed that the levels of 6-keto-PGF_1α_ and AA were significantly decreased (*p* < 0.05), whereas the levels of TXB_2_, 8-iso-PGF_2α_, PGF_2α_, 12S-HETE, 15S-HETE, 9S-HODE, and 13S-HODE were significantly increased in the MOD group compared to the CON group (*p* < 0.05), indicating that the HELP diet changed the AA metabolites. The levels of 6-keto-PGF_1α_ and AA were significantly increased (*p* < 0.05), while the levels of TXB_2_, LTD_4_, 8-iso-PGF_2α_, PGF_2α_, 12S-HETE, 15S-HETE, 9S-HODE, and 13S-HODE were significantly decreased in the BUL group compared to the MOD group (*p* < 0.05), indicating that the changes in AA metabolism induced by the HELP diet were reversed by *B. uniformis*. Notably, the levels of 6-keto-PGF_1α_ and AA were significantly increased (*p* < 0.05), and 15S-HETE was significantly decreased in the BUL group compared to the CON group (*p* < 0.05) (Figures 6A–N).

### 3.7 Correlations between gut microbiota, serum lipids, and AA metabolites

To further elucidate the relationship between differential bacteria, serum lipids, and AA metabolites, *Bacteroides*, *Treponema*, and *Helicobacter* were selected for Spearman’s correlation analysis. The results are shown in Figure 6O. The three differential bacterial genera were significantly correlated with serum lipid levels and AA metabolites. *Bacteroides* was significantly positively correlated with HDL-C, 6-keto-PGF_1α_, and AA (*p* < 0.05). Conversely, it showed a significant negative correlation between TG, TC, LDL-C, ALT, LTD_4_, 8-iso-PGF_2α_, PGF_2α_, and 15S-HETE (*p* < 0.05), indicating that *Bacteroides* has beneficial effects on improving FLHS in Dawu Golden Phoenix laying hens. On the other hand, *Treponema* exhibited a significant positive correlation with TG, TC, LDL-C, AST, ALT, TXB_2_, LTD_4_, PGE_2_, 8-iso-PGF_2α_, PGF_2α_, 12S-HETE, 9S-HODE, and 15S-HETE (*p* < 0.05). Conversely, it showed a significant negative correlation between HDL-C, 6-keto-PGF_1α,_ and AA (*p* < 0.01). Furthermore, *Helicobacter* exhibited a significant positive correlation between TG, TC, LDL-C, AST, ALT, TXB_2_, LTD_4_, 8-iso-PGF_2α_, PGF_2α_, 13S-HODE, and 15S-HETE (*p* < 0.05). Conversely, it showed a significant negative correlation between HDL-C, 6-keto-PGF_1α_, and AA (*p* < 0.01). These results indicate that *Treponema and Helicobacter* can aggravate FLHS.

## 4 Discussion

In prior investigations regarding probiotics, especially those focusing on intestinal flora regulation and metabolism improvement, numerous researchers have used comparable dosage levels. Li et al. administered *Lactiplantibacillus plantarum* FRT4 to laying hens at a dosage range of 1 × 10^9^–1 × 10^11^ CFU/ml. They found that it enhanced laying performance and mitigated FLHS induced by a HELP diet by regulating the gut–liver axis (Li et al., 2024). This provides a valuable reference for selecting *B. uniformis* dosages.

The stress resistance experiment indicated that *B. uniformis* was capable of growing and surviving at low pH and in the presence of bile salts, which demonstrated that *B. uniformis* could tolerate gastrointestinal environmental conditions. The Dawu Golden Phoenix laying hen, autonomously cultivated by China, represents an outstanding breed of high-yielding, red-feathered, pink-shell egg-laying hens. FLHS frequently afflicts commercial caged laying hens, particularly those with high production rates and excessive conditioning, and leads to mortality rates as high as 70% (Shini et al., 2018). *B. uniformis* and *Bacteroides xylanisolvens*, along with other Bacteroides species, have been reported to confer beneficial effects on NAFLD (Qiao et al., 2020; Lee et al., 2021). However, the role of *B. uniformis* in the development of FLHS in poultry is not yet clear. In our study, *B. uniformis* was isolated from the cecum of laying hens and administered to laying hens with FLHS caused by the HELP diet. Furthermore, our findings indicated that *B. uniformis* could ameliorate the adverse effects of FLHS by modulating the hepatic lipid metabolism, regulating gut microbiota, and improving AA metabolism.

The HELP diet is a widely adopted approach for inducing FLHS in chicken models (Miao et al., 2024). Prior studies have established that the HELP diet leads to hepatic lipid overload, thereby inducing lipotoxicity and initiating a cascade of cytotoxic events (Meng et al., 2021). In our experiment, the liver of the MOD group appeared yellowish-brown and greasy, with hemorrhages and bleeding spots. The liver lipid indicators, including TG, TC, LDL-C, ALT, AST, and abdominal fat rate, were significantly increased, which, combined with HDL-C, were significantly reduced in the MOD group. These results confirmed the successful establishment of the FLHS model in laying hens, consistent with existing literature (Li et al., 2024). Notably, the adverse effects observed in the MOD group were significantly reversed by *B. uniformis* treatment. It is worth mentioning that the reversal effect of BUL on the liver index and the levels of LDL-C, HDL-C, ALT, and AST was better than that of BUH. This conclusion has also been confirmed by the macroscopic images and the pathological changes in the liver. These findings were consistent with those of a previous study reporting that *B. uniformis CECT 7771* could reduce serum cholesterol and triglyceride levels in obese mice and alleviate the metabolic and immune disorders caused by high-fat diets (Fabersani et al., 2021).

Impaired intestinal barrier function, increased intestinal permeability, altered bile acid metabolism, and endotoxin-activated hypo-inflammation are all linked to the disrupted intestinal flora, which are involved in the development and progression of FLHS (Dansou et al., 2024). It has been shown that the relative abundance of Bacteroidota is reduced in FLHS-laying hens (Liu et al., 2022). *Bacteroides* has also been demonstrated to promote hepatic glucose storage and influence selective intestinal lipid absorption and serum triglyceride levels (López-Almela et al., 2021). Moreover, rats treated with atorvastatin exhibited a higher abundance of *Bacteroides* in the gut, which led to a reduction in blood lipid levels, including those of TG, TC, and LDL-C. This occurred through the enhancement of propanoate metabolism as well as the metabolism of glycine, serine, and threonine in both feces and plasma, thereby contributing to the overall reduction of blood lipid levels (Li et al., 2022). In our experiments, there was a significant decrease in the relative abundance of Bacteroidota in the MOD group. Supplementing *B. uniformis* increased the relative abundance of *Bacteroides*. *Bacteroides* was significantly negatively correlated with AST, TG, TC, and LDL-C levels (Yang et al., 2023; Zhang et al., 2024a, 2024b). In our experiments, *Bacteroides* was positively correlated with HDL-C and negatively correlated with TG, TC, LDL-C, and ALT. These results were consistent with the previous findings that the increased abundance of *Bacteroides* could reduce the levels of TG, TC, and LDL-C and increase the level of HDL-C in HFD-fed mice (Peng et al., 2023). In addition, our results showed that *Bacteroides* levels also exhibited a positive correlation with 6-keto-PGF1α and AA and a negative correlation with LTD_4_, 8-iso-PGF_2α_, PGF_2α_, and 15S-HETE, suggesting that an increase in *Bacteroides* abundance may be linked to the metabolic process of AA.

TXB_2_ is a metabolite of thromboxane A_2_ (TXA_2_). In lipid metabolism, elevated levels of TXA_2_/TXB_2_ are frequently associated with dyslipidemia and atherosclerosis (DeFilippis et al., 2014; Petrucci et al., 2024). Prostaglandins (PGs) like PGF_2α_ and 8-iso-PGF_2α_ are involved in the inflammatory process, which could disrupt normal lipid-handling mechanisms (Wentzel and Eriksson, 2002; Wang et al., 2021). Leukotrienes, like LTD_4_, are also inflammatory mediators. They could exacerbate inflammation in adipose tissue and the liver, potentially interfering with normal lipid metabolism pathways (Ma et al., 2024). Some studies have reported that *Alox15* deficiency, which lowers plasma 15-HETE levels, protects against steatohepatitis, hyperlipidemia, and alcoholic liver disease by reducing reactive oxygen species production, hepatic steatosis, insulin resistance, and inflammatory injury (Yang et al., 2024). 6-keto-PGF_1α_ is a stable metabolite of prostaglandin I_2_ (PGI_2_) (Griffith et al., 2022). PGI_2_ is widely recognized for its vasodilatory and anti-platelet aggregation properties (Rogula et al., 2020). In Cav-1 knockout endothelial cells (Cav-1 KO EC), there is autocrine production of PGI_2_. This increase in PGI_2_ stimulates the cAMP/PKA pathway, thereby contributing to enhanced lipolysis in Cav-1 KO ECs (Kuo et al., 2017). AA, a crucial omega-6 fatty acid, is a precursor to a suite of PGs and LTB_4_. As potent inflammatory mediators, AA plays an important role in inflammation, lipid metabolism, and the regulation of energy balance (Pereira et al., 2024). Our previous research demonstrated that AA levels tend to decrease with the administration of high-fat diets in broilers (Meng et al., 2021). Dietary supplementation with omega-6 polyunsaturated fatty acids, including AA, was shown to favorably affect plasma LDL-C and HDL-C, the AA metabolome, as an important regulator of cholesterol homeostasis (Demetz et al., 2014). AA is activated by the cyclooxygenase (COX) pathway to produce PGG₂, PGH₂, TXB₂, and PGE₂. PGH₂ generates PGF2α and PGI₂ under the catalysis of different enzymes (Xu et al., 2021; Xie et al., 2024). Our study showed that *Bacteroides* could affect the AA metabolism through COX pathways.

In metabolic syndrome, which includes a series of diseases such as obesity, hypertension, and dyslipidemia, the Firmicutes/Bacteroidetes ratio plays an important role in the occurrence and progression of these diseases (Stojanov et al., 2020). A higher ratio of Firmicutes/Bacteroidota disrupted energy homeostasis, increased lipid synthesis, caused chronic low-grade inflammation, and led to obesity (Ridlon et al., 2006; Zhang et al., 2009; Yun et al., 2017; Cuevas-Sierra et al., 2019). In our experiments, the ratio of Firmicutes/Bacteroidota was significantly increased in the MOD group and reduced after *B. uniformis* treatment. Spirochaeta is known to induce gastrointestinal inflammation (He et al., 2023). Taurine could significantly ameliorate LPS-induced intestinal injury through a decrease in Spirochaetota in the colon (Zhao et al., 2023). In our study, the relative abundance of Spirochaetota in the MOD group was significantly increased and decreased after *B. uniformis* treatment.

*Helicobacter* was a genus consisting of many pathogenic bacteria associated with gastric inflammation and inflammatory bowel disease (Shibata et al., 2017; Schroeder et al., 2018). A lower relative abundance of *Helicobacter* was considered beneficial for ameliorating metabolic endotoxemia in obese mice (Luo et al., 2022). *Treponema* is a spirochetal bacterium that can colonize a wide range of hosts and tissues, causing different clinical symptoms (Dias et al., 2024). In our study, the supplementation of *B. uniformis* led to a concurrent decrease in the relative abundance of *Treponema and Helicobacter*. Therefore, we speculated that *Treponema* and *Helicobacter* could affect the AA metabolism through the LOX and COX pathways.

In microbial intervention studies, high-dose probiotics are not always more effective than low-dose probiotics, which may be related to multiple factors. The interaction between intestinal microbes and the host immune system is complex. Moderate microbial stimulation helps maintain immune homeostasis, whereas excessive stimulation may cause immune disorders and affect the disease-modifying effects of probiotics (Round and Mazmanian, 2009). In the present study, the high-dose *B. uniformis* (BUH group) induced immune overactivation and interfered with the regulation of liver index and lipid parameters, resulting in less effective treatment. The stability and balance of a microbial community are crucial for its functions. A massive influx of foreign microorganisms may disrupt the original balance (Costello et al., 2009). In the BUH group, the excessively high doses of *B. uniformis* may compete with other microorganisms in the gut for nutrients or living space. Moreover, the metabolic products it produces may also inhibit its growth by itself or other beneficial bacteria, thereby affecting the overall regulatory effect on liver index and blood lipid parameters.

In this study, we found significant correlations among *Bacteroides*, *Treponema*, *Helicobacter*, serum lipids, and AA metabolites. Regarding the limitations of correlation analysis, it is indeed impossible to clearly infer causal relationships solely through Spearman correlation analysis. Fecal microbiota transplantation is a very promising research method. By transplanting a specific microbiota from one individual to another, it is possible to more directly observe the impact of microbiota changes on aspects such as host metabolism and disease development, thus helping to determine the causal relationship between the microbiota and host health (Almeida et al., 2022). We plan to actively adopt this method in subsequent research to deeply explore the potential mechanisms by which *B. uniformis* improves FLHS, make up for the shortcomings of this study in inferring causal relationships, and provide a more in-depth and comprehensive theoretical basis for research in this field.

## 5 Conclusion

Collectively, the HELP diet resulted in a lower egg production rate and feed conversion ratio and accelerated FLHS formation and development in laying hens. Supplementation of *B. uniformis* significantly increased egg production rate and feed conversion ratio and relieved FLHS with the decline of TG, TC, LDL-C, AST, ALT, and the increase of HDL-C in the liver. Importantly, *B. uniformis* reshaped gut microbiota structure and AA metabolism caused by the HELP diet. In summary, *B. uniformis* mitigated FLHS in laying hens through regulating liver function, gut microbiota, and AA metabolism. Therefore, supplementation of *B. uniformis* could be an effective way to reduce FLHS in laying hens.

## Data Availability

The data presented in the study are deposited in the National Center for Biotechnology Information (NCBI) BioProject database repository, accession number PRJNA1247204.

## Footnotes

^1^ https://www.NCBI.nlm.nih.gov/
