# Supplementary material for: Impact of bacteroides uniformis on fatty liver hemorrhagic syndrome in dawu golden phoenix laying hens: modulation of gut microbiota and arachidonic acid metabolism
Source: Front Microbiol. 2025 Apr 28;16:1560887. doi: 10.3389/fmicb.2025.1560887 (PMC12066428; doi:10.3389/fmicb.2025.1560887)
Supplement: Supplementary file 1 [file Data_Sheet_1.docx]

Impact of *Bacteroides uniformis* on Fatty Liver Hemorrhagic Syndrome in Dawu Golden Phoenix Laying Hens: Modulation of Gut Micorbiota and Arachidonic Acid Metabolism

Yu Zhang^1^, Rongfei Ma^1^, Xicui Du^2^, Xin He^1^, Yan Zhang^3^, Ning Ma^1^, Hailong Liu^3^*, Xinghua Zhao^1^*

^1^ College of Veterinary Medicine, Hebei Agricultural University, Baoding, Hebei, 071000, China.

^2^ Hebei Jinkun Animal Pharmaceutical Co. Ltd., Xinji, Hebei, 052360, China

^3^ Institute of Animal Science and Veterinary Medicine, Hainan Academy of Agricultural Sciences, Haikou, 571100, China.

* Corresponding author:

Xinghua Zhao, Address: No.2596, Lekai South Street, Baoding, 071000, China

E-mail: [dyzhaoxh@hebau.edu.cn](mailto:dyzhaoxh@hebau.edu.cn)

Hailong Liu, Address: No.14, Xingdan Road, Haikou, 571100, China

E-mail:[Liuhailong423@126.com](mailto:Liuhailong423@126.com) (Hailong Liu)

Supplementary Materials

*Bacterial strains and culture conditions*

The *B. uniformis* strain was cultured in anaerobically sterilized Brain Heart Infusion (BHI) broth containing 0.01% hemin, 0.01% vitamin K1, and 0.01% L-cysteine (Qingdao Hi-Tech Industrial Park Hope Bio-Technology Co., Ltd., Shandong, China) at 37℃ for 24 h. The *B. uniformis* culture medium was centrifuged at 8000×g for 10 minutes at 4℃. The bacterial sludge was then washed twice with sterile PBS and resuspend to 1 × 10^11^ CFU/mL for animal experiment (Zhang et al., 2024).

*Acid, bile and simulated gastrointestinal fluid tolerance*

The acid, bile and gastrointestinal fluid tolerance of *B. uniformis* were checked according to the methods described in previous literature with minor modifications (Liu et al., 2021; Yang et al., 2022; Zhang et al., 2022; Kouadri Boudjelthia et al., 2023). Specifically, the overnight cultured strain at 37 °C for 24 h was harvested by centrifugation at 4 °C, 4000×g for 10 minutes. the activated of *B. uniformis* were the final precipitate which was washed three times using phosphate-buffered saline (PBS, pH 7.2). Acid tolerances were performed in BHI broth and adjusted to a final pH was 3 using 1 mol/L HCl. The activated *B. uniformis* was added into the acid BHI broth and cultured anaerobically at 37 °C for 3 h. Simulated gastrointestinal fluid and simulated gastric acid tolerance were performed as follows: the activated *B. uniformis* were resuspend with simulated gastric juice or simulated gastric acid and incubated at 37 °C for 4 h. Bile tolerance was examined as follows: 0.2 mL of the activated *B. uniformis* was cultured anaerobically into 9.8 mL in BHI broth with 0.1%, 0.3%, and 0.5% (w/v) sodium taurocholate (Sangon Biotech Co., Ltd., Shanghai, China) and incubated at 37 °C for 12 h. Then the survival rates (%) were calculated as the percentage of the number of viable bacteria grown on BHI broth or simulated gastrointestinal fluid after incubation (N1, CFU/mL) and the initial number of viable bacteria (N0, CFU/mL) according to Eqs. (1) (Liu et al., 2021).

$\text{Survival rate (\%) = }\frac{\boldsymbol{N}_{\mathbf{1}}}{\boldsymbol{N}_{\mathbf{0}}}\boldsymbol{*100\%}$ (1)

Table S1. Composition and nutrient levels of the standard diet and HELP diet (air-dry basis, %).

| Item | Standard diet | HELP diet |
| --- | --- | --- |
| Ingredients |  |  |
| Corn | 64.00 | 70.00 |
| Wheat bran | 2.00 | 1.20 |
| Soybean meal (44% crude protein) | 24.00 | 14.58 |
| Fat-soybean oil |  | 4.22 |
| Calcium carbonate | 8.00 | 8.00 |
| Premix^1^ | 2.00 | 2.00 |
| Total | 100.00 | 100.00 |
| Nutrient levels |  |  |
| Crude protein^2^ | 15.80 | 12.30 |
| Phosphorus^2^ | 0.54 | 0.51 |
| Calcium^2^ | 3.55 | 3.53 |
| Lysine^3^ | 0.96 | 0.69 |
| Arginine^3^ | 1.03 | 0.74 |
| Methionine^3^ | 0.37 | 0.32 |
| Valine^3^ | 0.77 | 0.58 |
| Metabolic energy, kcal/kg^3^ | 2679 | 3100 |
| Met + Cys^3^ | 0.67 | 0.56 |

^1^ The ingredient of premix per gram: VA, 12,500 IU; VD_3_, 32,500 IU; VE, 18.75 mg; VK_3_, 2.65 mg; VB_1_, 2 mg; VB_2_, 6 mg; VB_12_, 0.025 mg; CaHPO_4_, 500.00 mg; cupric sulfate, 4.6 mg; ferrous sulfate, 28.4 mg; manganous sulfate, 35.46 mg; zinc sulfate, 76 mg; zeolite powder, 6 mg; sodium selenite, 5 mg; anti-oxidizing quinolone, 50 mg; choline, 90 mg; bacitracin zinc, 26.7 mg; methionine,100 mg.

^2^ All data results were obtained through chemical analysis and were presented as the average of two measurements.

^3^ The levels of amino acids and metabolic energy were calculated based on the Tables of Feed Composition and Nutritive Values in China (31st edition).

The used feed was commercial diet. The formulation of standard diet followed the guidelines of the Chinese Feeding Standard of Chickens NPC (1994). The determination of crude protein in feed followed the Chinese national standard (GB/T 6432-2018) using the Kjeldahl method. The automatic Kjeldahl nitrogen analyzer employed in this process was purchased from FOSS Company (Hilloeroed, Denmark). For the measurement of total phosphorus in feed, the spectrophotometric method as outlined in the Chinese national standard (GB/T 6437-2018) was employed. The sample preparation for the feed was carried out by dry-ashing, followed by the addition of ammonium vanadomolybdate reagent for reaction. Subsequently, the absorbance was measured at 400 nm using a UV-visible spectrophotometer (SHIMADZU, Kyoto, Japan). The phosphorus content is then calculated by comparing the absorbance values to a standard curve. Finally, the calcium content in the feed was determined according to the Chinese national standard (GB/T 6436-2018).

Table S2. Egg production, egg weight, liver weight, and abdominal fat weight.

| Item | CON | MOD | BUL | BUH | SEM | *P*-value |
| --- | --- | --- | --- | --- | --- | --- |
| Egg production | 66.35^a^ | 53.13^c^ | 63.33^ab^ | 61.98^b^ | 0.822 | *P* < 0.001 |
| Egg weight (g) | 59.51^a^ | 56.29^b^ | 60.19^a^ | 58.93^a^ | 0.485 | *P* = 0.012 |
| Liver weight (g) | 37.58^a^ | 47.72^b^ | 37.34^a^ | 41.11^a^ | 0.828 | *P* < 0.001 |
| Abdominal fat weight (g) | 45.69^a^ | 98.55^b^ | 44.86^a^ | 51.75^a^ | 3.436 | *P* < 0.001 |

Means with different superscripts differ significantly at a significance level of *P* < 0.05, n = 12.

^1^Data are expressed as mean and SEM.


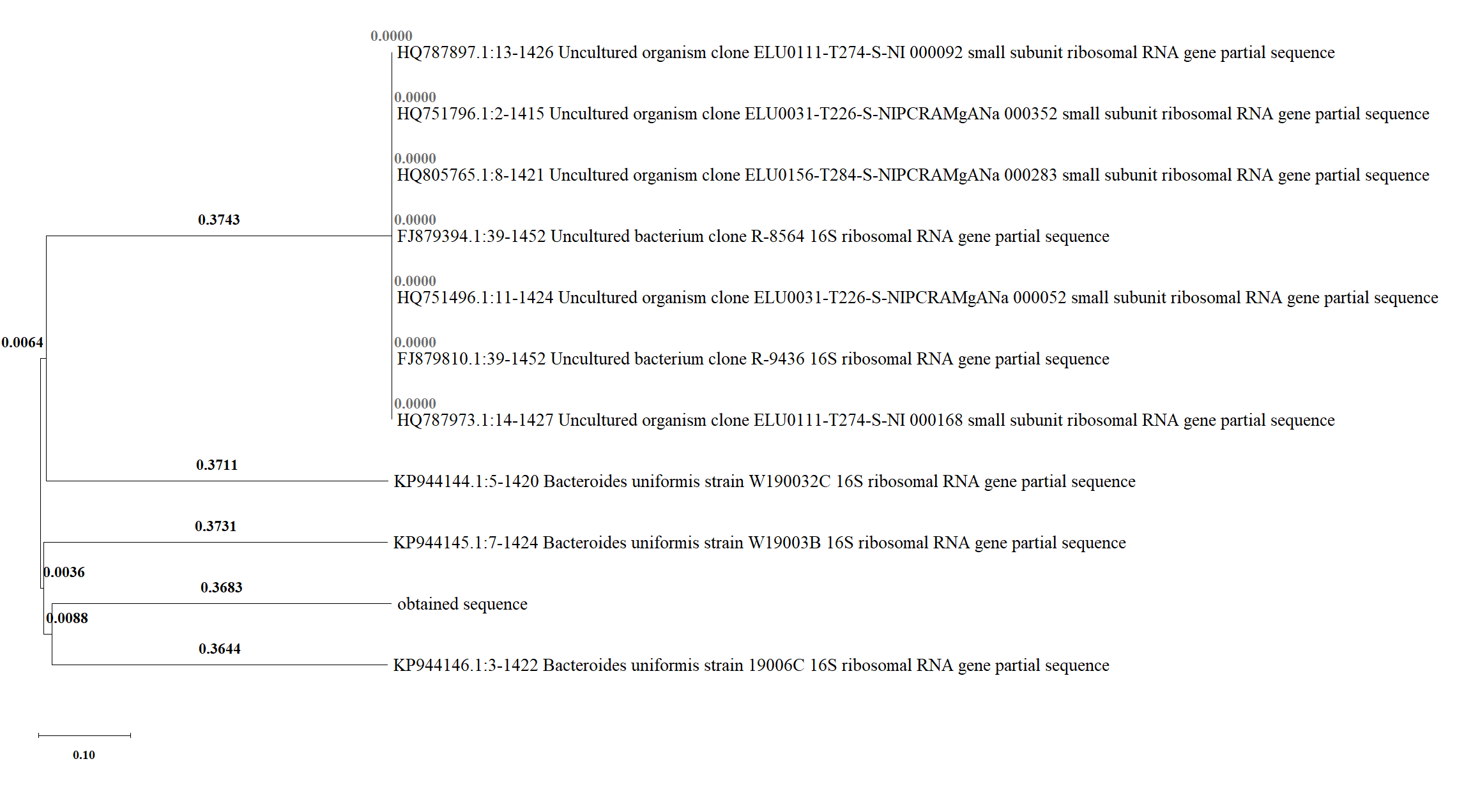
 Figure S1. Phylogenetic evolutionary tree of *Bacteroides*

References

Kouadri Boudjelthia, N., Meryem Belabbas, M., Bekenniche, N., Monnoye, M., Gérard, P., and Riazi, A. (2023). Probiotic Properties of Lactic Acid Bacteria Newly Isolated from Algerian Raw Cow’s Milk. *Microorganisms.* 11, 2091. doi: 10.3390/microorganisms11082091

Liu, C., Han, F., Cong, L., Sun, T., Menghe, B., and Liu, W. (2021). Evaluation of tolerance to artificial gastroenteric juice and fermentation characteristics of *Lactobacillus* strains isolated from human. *Food Sci Nutr.* 10, 227-238. doi: 10.1002/fsn3.2662

Yang, M.-F., Yan, W., Li, Y., Li, S.-Q., Chen, H.-Y., Yin, Q.-Q., et al. (2022). The Probiotic Attributes and Anti-pseudorabies Virus Evaluation of Lactobacillus Isolates. *Front Vet Sci.* 9, 902052. doi: 10.3389/fvets.2022.902052

Zhang, L., Qu, H., Liu, X., Li, Q., Liu, Y., Wang, W., et al. (2022). Comparison and selection of probiotic *Lactobacillus* from human intestinal tract and traditional fermented food in vitro via PCA, unsupervised clustering algorithm, and heat‐map analysis. *Food Sci Nutr.* 10, 4247-4257. doi: 10.1002/fsn3.3018

Zhang, S., You, M., Shen, Y., Zhao, X., He, X., Liu, J., et al. (2024). Improving fatty liver hemorrhagic syndrome in laying hens through gut microbiota and oxylipin metabolism by *Bacteroides fragilis:* A potential involvement of arachidonic acid. *Anim Nutr.* 20, 182-199. doi: 10.1016/j.aninu.2024.08.008
